# **The effects of graded hypoxia on gene expression related to oxygen sensing and metabolism**

**DOI:** 10.1038/s41598-025-31608-8

**Published:** 2025-12-17

**Authors:** Josey Walker, Brent Ruby, Dustin Slivka

**Affiliations:** School of Integrative Physiology and Athletic Training, College of Health, University of Montana, Missoula, MT USA

**Keywords:** PGC-1α, HIF-1α, Normobaric, Recovery, Simulated altitude, Medical research, Physiology

## Abstract

To improve performance at altitude some endurance athletes undergo exposure to hypoxic stimulus during post exercise recovery, but this may impact gene expression related to exercise adaptation. The purpose of this study is to determine a hypoxic threshold and its impact on gene expression related to oxygen sensing, metabolism, and mitochondrial dynamics. Ten male participants (24 ± 4 years, 47.1 ± 9.5 ml·kg^− 1^·min^− 1^ ) completed 4 trials at least 7 days apart. Each trial consisted of cycling for one hour at 70% of Wpeak in normoxia before recovering for 6 h in a simulated environment [0 m, 1,667 m, 3,333 m, and 5,000 m]. Muscle samples were collected from the *vastus lateralis* before exercise and after recovery. Pulse oxygen saturation (SPO_2_) was measured before exercise, during exercise, and during recovery. SPO_2_ lowered with increasing elevation (*p* < 0.001 for all trials). Gene expression of PGC-1α, HIF-1α, and HK increased following recovery from exercise (*p* = 0.048, *p* = 0.013, and *p* = 0.014), but there were no differences between trials (*p* > 0.05). No differences were observed for HIF-2α, PFK, FIS, MFN 2, and OPA (*p* > 0.05). Post aerobic exercise recovery at graded levels of simulated hypoxia does not affect the expression of genes related to oxygen sensing, metabolism, and mitochondrial dynamics, compared to normoxia.

## Introduction

Adaptations to exercise that are necessary for improved performance begin through acute signaling that leads to gene transcription and expression^1,2^. This signaling cascade can be impacted by a variety of factors such as nutrition, hydration status, training status, and environment^3–7^. If gene expression related to mitochondria is suppressed it could lead to the reduction of training adaptations that endurance athletes strive for^2^. Hypoxia has been shown to have a nonlinear dose-response relationship with many aspects of human physiology, such as submaximal exercise performance, aerobic capacity, and muscle sympathetic nervous activity^8–11^. Typically decrements become increasingly severe with elevation gain. Previous research on well-trained males has demonstrated that an acute 6 h exposure to simulated altitude, 5,000 m, following aerobic exercise may limit gene expression of Hypoxia Inducible Factor (HIF) -1α, HIF-2α, Peroxisome Proliferator-activated receptor gamma coactivator 1α (PGC-1α), Mitofusin 2 (MFN2), and Optic Atrophy Gene 1 (OPA). Fold change of these genes was observed to be as low as 0.5 times fold change in normoxic recovery^12^. These genes are involved in oxygen sensing by the cell and the dynamics of mitochondrial changes. However, this blunting has not been demonstrated in studies investigating single simulated altitudes below 4,200 m, potentially indicating a threshold of hypoxic stimulus^13,14^.

When competing at altitude, performance is reduced compared to sea level due to limited oxygen availability secondary to the reduced partial pressure of oxygen. Endurance athletes use altitude training to try and mitigate this reduction in oxygen availability’s impact on performance^9^. When acclimatized to high elevation the body undergoes adaptive changes to live at altitude including decreased mitochondrial density, reduced vascular blood flow, and a cachectic response^15–19^. While these adaptations appear detrimental, they prioritize oxygen transport and transit time. For example, some evidence indicates that despite decreased mitochondrial density, the organelle’s oxidative metabolism becomes more efficient^17,20^. Improved efficiency of the mitochondrial reticulum could explain improved endurance performance following acclimatization, at sea level and altitude, when compared to non-acclimatized individuals^9,10,21–28^. The traditional method of altitude training requires the athlete to live and train at altitude for approximately 2 weeks before their event ‘Live High - Train High’^29^. However, there is controversy over whether Live High - Train High provides benefits to sea level performance^30^ and there is still uncertainty surrounding the performance benefit of altitude training in general^31^.

In contrast, some endurance athletes choose to recover within a normobaric hypoxic environment but train in a normoxic environment to preserve exercise workload, referred to as ‘Live High - Train Low’^29^. Other methods of acclimation utilized by athletes are the traditional ‘Live High - Train High’, and ‘Live Low – Train High’. Live High – Train High is typically achieved by the athlete travelling to the altitude they will be competing at approximately 2 weeks before competition. Where they will maintain their training regimen and naturally acclimate. Live Low – Train High involves athletes either entering simulated hypoxia for training only or staying in an artificially normoxic environment and exiting to train at their competition altitude^29^. Maintaining a sea level training load in combination with exposure to hypoxia during recovery periods in theory allows the athlete to improve sea level performance while acclimating. Endurance athletes undergoing Live High – Train Low acclimation protocols in simulated altitude are recommended to spend 12–16 h per day between 2,500 m and 3,000 m. Simulated altitude is achieved through reducing the fraction of inspired oxygen in the environment. Many endurance athletes choose to sleep in a hypoxic tent to meet these requirements. This technique appears to be advantageous over other methods of altitude acclimation for sea level performance^30,32^. Some individuals may experience sleep disruptions at altitude^33,34^. Adaptations from acclimatization may be achieved through increased red blood cell volume, and oxygen utilization^35^ or improved economy and buffering capacity^36^ avoiding negative impact on the mitochondria^37^. However, less is known about how acute exposure to hypoxia during recovery impacts the cellular response to exercise stimulus. Given our understanding of chronic hypoxic adaptations this study may provide insight into how acute cellular response informs chronic adaptations.

Endurance athletes compete in a myriad of environmental conditions, from sea level to high altitude. Chronic exposure to high altitude appears to decrease mitochondrial density^15,16,18,19^ which is counter to aerobic exercise performance needs. Similarly, at simulated elevations up to 5,000 m, an acute hypoxic exposure causes blunted mitochondrial gene expression^12^, suggesting that this acute signaling explains chronic mitochondrial outcomes. The level of hypoxic stimulus at which gene expression is limited has yet to be elucidated fully, as well as the implications of this acute limitation on long term adaptations^12–14^. It is important to investigate whether ‘Live High – Train Low’ provides suboptimal stimulus for exercise adaptation for endurance athletes^2^. The purpose of this study is to investigate the impact of graded normobaric hypoxic exercise recovery environments [0 m, 1,667 m, 3,333 m, and 5,000 m] on the gene expression of proteins involved in oxygen sensing (HIF-1α, HIF-2α), metabolism (Phosphofructokinase (PFK), Hexokinase (HK)), and mitochondrial dynamics (PGC-1α, mitochondrial fission 1 protein (FIS-1), OPA, and MFN 2). We hypothesize there will not be a limiting effect on gene expression during recovery from aerobic exercise at simulated altitudes below 5,000 m.

## Methods

### Participants

Ten non-obese recreationally active college aged male participants were recruited from the university and surrounding area. Participants were 24 ± 4 years old, 182.8 ± 4.6 cm tall and weighed 86.0 ± 13.6 kg. Average BMI was 25.7 ± 3.9 with a body fat percentage of 15.9 ± 7.1%. Aerobic fitness assessed through VO_2_peak which averaged 4.0 ± 0.5 L·min ^− 1^, or 47.1 ± 9.5 ml·kg^− 1^·min^− 1^, and was associated with a Wpeak of 287 ± 42 W. All participants resided at an altitude of approximately 978 m and had not been exposed to elevations greater than 2,000 m within the last 3 months. Given our participants were only acclimated to altitude below our experimental conditions we do not believe previous acclimation status impacted our findings. Participants completed a Physical Activity Readiness Questionnaire^38^ and were briefed on the experimental protocol and possible risks prior to providing written informed consent. To ensure homogeneity of response without the influence of hormonal fluctuations related to the menstrual cycle in this initial proof of concept study female participants were excluded. All procedures were approved by the University of Montana Institutional Review Board and performed in accordance with the declaration of Helsinki.

### *Preliminary testing* 

Body composition was calculated using hydrodensitometry. Underwater mass was measured with a digital scale (Exertech, Dreshbach, MN). Body density was corrected for estimated residual lung volume^39^ and converted to percent body fat using the Siri equation^40^. A graded maximal exercise test (starting at 95 W, and increasing 35 W every 3 min)^12–14,41^ was completed on an electronically braked cycle ergometer (Velotron, RacerMate Inc., Seattle, WA) to determine aerobic capacity (VO_2_peak) and associated aerobic power output (Wpeak). Gas analysis was completed in 15 s intervals utilizing a calibrated metabolic cart (ParvoMedics, Inc., Salt Lake City, UT). VO_2_peak was assigned to the highest achieved oxygen uptake recorded during the test. Wpeak was calculated by adding the power output in the last completed stage to the fraction of time spent in the uncompleted stage multiplied by 35. Percentage of Wpeak associated is VO_2_peak has been shown to be a reasonable approximation of percent VO_2_peak.

$$\:{W}_{max}=\left(Power\:\left(W\right)\:of\:final\:completed\:stage\right)+\left(\frac{time\:in\:final\:stage\:before\:fatigue\:\left(s\right)}{stage\:time\:\left(180\:s\right)}*35W\right)$$

### Experimental Protocol

Participants replicated diet and exercise before each trial by keeping an exercise record for 2 days before and a dietary record for 24 h before the initial trial. Additionally, they abstained from exercise, caffeine and alcohol for 24 h before each trial. Each participant completed all four trials in a randomized counter-balanced order. Following an overnight 12 h fast, participants arrived at the laboratory in the early morning to complete testing. A total of 4 trials were completed per participant. Participants performed a 60-minute cycling protocol at ~ 70% of Wpeak attained during VO_2_peak test. Upon completion of exercise participants were allowed to change before entering the environmental chamber for recovery. Then they remained in a sitting position throughout the 6-hour recovery period in one of four normobaric simulated environments [0 m, 1,667 m, 3,333 m, and 5,000 m]. Snack bars and a piece of fruit were provided (CLIF^®^ and/or PowerBar^®^, and an apple and/or banana) (1.2 g·kg^− 1^ carbohydrate, 0.15 g·kg^− 1^ fat, and 0.29 g·kg^− 1^ protein) at 0 and 3 h into recovery. Water was consumed *ad-libitum* during the ride and the recovery; hydration status was not controlled between trials. Visits occurred in a randomized, counterbalanced cross-over design over the span of 4 weeks, with a minimum of 7 days between trials. The use of randomized counterbalanced trials in combination with the wash out period should control for possible carryover effect. Due to our methodology, we do not anticipate an impact of altitude exposure on overall results. All trials were completed at a terrestrial altitude of 978 m in a temperature, humidity, and hypoxia (Colorado Altitude Training, Louisville, CO) controlled environmental chamber (Tescor, Warminster, PA) set at a constant 22 °C and 40% relative humidity. The fraction of inspired oxygen was adjusted to account for the testing altitude of 978 m and was maintained by the environmental chamber. Thus, the fraction of inspired oxygen was 25.7% O_2_ at 0 m, 18.9% at 1,667 m, 15.3% at 3,333 m, and 12.8% at 5,000 m.

### Biopsies

Muscle biopsies were taken before exercise, and at the end of the 6-hour recovery period for each trial. Biopsies were taken from the *vastus lateralis* using a 5 mm Bergstrom percutaneous muscle biopsy needle with the aid of suction^42^. The biopsy area was sterilized with Povidone Iodine (10%) swabs, and locally anesthetized with Lidocaine HCl (1%) via subcutaneous and intramuscular injection. Following anesthetization, a small (~ 0.6 cm) incision was made in the skin allowing tissue collection. All subsequent biopsies during a given trial were obtained from the same leg using a separate incision 2 cm proximal to the previous biopsy. Incisions were closed with a Steri-Strip and covered with a sterile adhesive bandage; direct pressure was applied to the site with gauze under an elastic Tensoplast (BSN Medical, South Africa) strip on top of the bandage to reduce bruising. Excess blood, connective tissue, and fat were immediately removed from tissue samples and were stored in RNA Later at −80 °C for later analysis.

### Pulse oximetry

Pulse oxygen saturation (SPO_2_) (Nonin Onyx II 9550, Plymouth, MN) was measured before exercise. SPO_2_ during exercise was taken 45 min into exercise, and immediately before exercise cessation. These two measures were then averaged to represent exercise SPO_2_. During recovery, SPO_2_ was measured hourly at recovery times: 0 min, 60 min, 120 min, 180 min, 240 min, 300 min, 355 min), the grand average of the seven recovery measurements was taken to represent the entire recovery period.

### Gene expression analysis

Skeletal Muscle RNA isolation: An 8–20 mg piece of skeletal muscle was homogenized in 800ul of Trizol using an electric rotor stator style homogenizer. The samples are then incubated at room temperature for 5 min after which 200 ul of chloroform per 1000 ul of Trizol was added and shaken vigorously by hand. After an additional incubation at room temperature for 2–3 min the samples were centrifuged at 12,000 g for 15 min and the aqueous phase was transferred to a fresh tube. The RNA was then precipitated by adding 500 ul of isopropyl alcohol per 1000 ul of initial Trizol and incubated overnight at −20 °C. The next morning samples were centrifuged at 12,000 g for 10 min at 4 °C and the RNA was washed by removing the supernatant and adding 1000 ul of 75% ethanol per 1000 ul of initial Trizol. The samples were then mixed by vortex and centrifuged at 7,500 g for 5 min at 4 °C. The RNA was then redissolved in 100 ul RNase-free water after the supernatant was removed and the RNA pellet was dried. The RNA was then cleaned using the RNeasy mini kit (Qiagen, catalog number 74104) according to the manufactures protocol using the additional DNase digestion step (RNase-free DNase set, Qiagen). RNA was then quantified using a nano-spectrophotometer (ND-1000, NanoDrop, Wilmington, DE).

### cDNA synthesis

First-strand cDNA synthesis was achieved using Superscript-fist strand kit (Invitrogen) according to the manufactures protocol. Each sample within a given subject was adjusted to contain the same amount of RNA (400–1000 ng). The resulting cDNA was then diluted using RNase free water to have adequate volume for RT-PCR and frozen for later RT-PCR analysis.

### Real time RT-PCR

Each 25 ul reaction volume contained 500 nM primers and 250 nM probe (PrimeTime qPCR assay, Integrated DNA technologies, Coralville, IA), 1x FastStart TaqMan Probe master (Roche Applied Science, Indianapolis, IN), and 2.5 ul of sample cDNA. PCR was run using the Bio-Rad I Cycler iQ5 Real-Time PCR Detection system (Bio-Rad, Hercules, CA) using a standard 2-step Roche protocol. Quantification of mRNA for genes of interest at 6-hours post-exercise was calculated using the 2^−ΔΔCT^ method^43^ relative to pre-exercise and the geometric mean of our 5 stable reference genes (B-actin, B2M, PPIA, GAPDH, and RPS18)^44^. These reference genes were used to ensure stability for the 2^−ΔΔCT^ method. If any reference gene of a given individual had a stability value of > 0.15 it was removed from analysis, and the geometric mean of the remaining reference genes was used. The 2^−ΔΔCT^ method of quantification does not follow a normal distribution and to account for this, statistical analyses were performed on log transformed data with all gene expression fold change values graphically presented on a normalized scale.

### Statistics

SPO_2_ and gene expression were analyzed using a repeated measure ANOVA (trial*time). In the event of a significant ANOVA, Fishers protected least significant difference method was used post-hoc to determine where differences occurred. A probability of type I error less than 5% was considered significant (*p* < 0.05). All data was tested for sphericity using Mauchley’s test of sphericity, if sphericity was violated a Greenhouse-Geisser correction was applied. A post hoc power analysis from SPO_2_ revealed an achieved power of 1.00. Descriptive data and SPO_2_ are reported as mean ± SD, while gene expression is reported as mean ± SE. Statistical data analysis was performed using the Statistical Package for Social Sciences (SPSS) software for Windows version 29 (IBM Corp., Armonk, NY; https://www.ibm.com/products/spss-statistics).

## Results

**Fig. 1:**
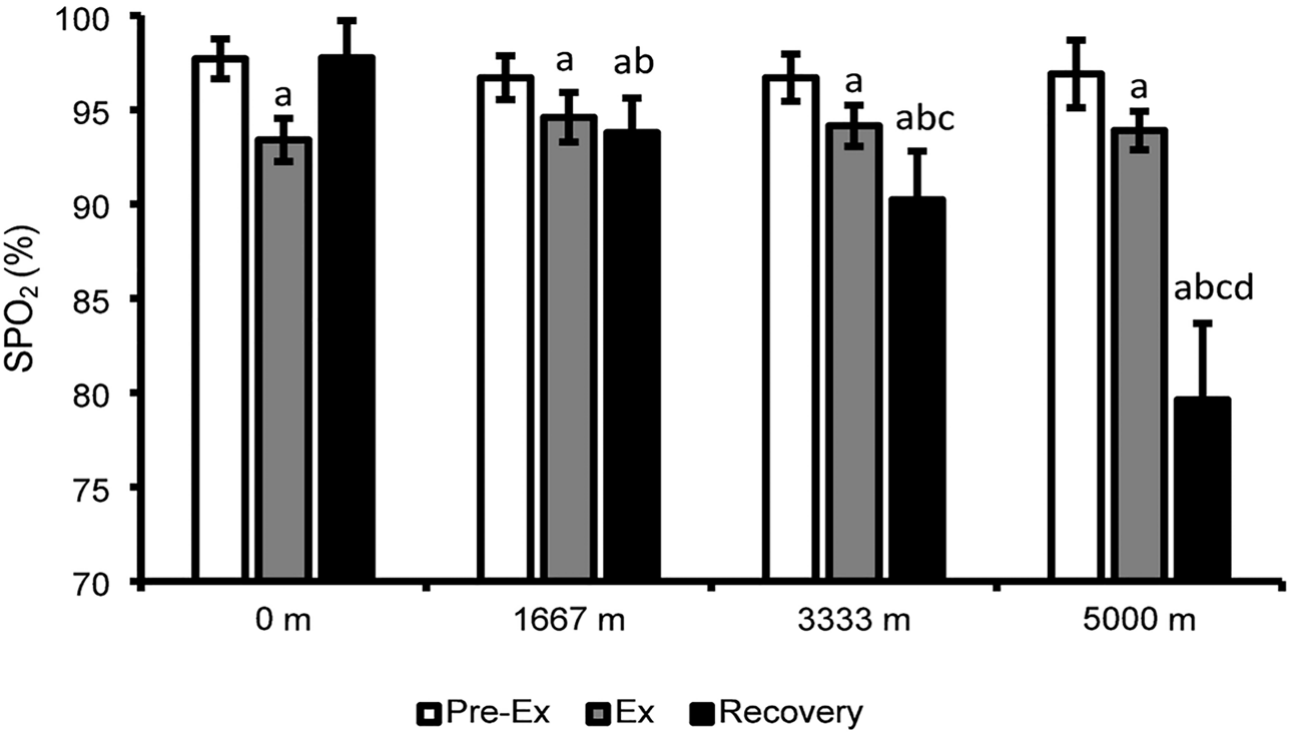
Average pulse oxygen saturation (SPO_2_) values before exercise (Pre-Ex), average during exercise (Ex), and average during the 6 h post-exercise recovery period (Recovery). (**a**) Denotes *p* < 0.05 compared to the pre-ex value within trial, (**b**) denotes *p* < 0.05 compared to 0 m recovery, (**c**) denotes *p* < 0.05 compared to 1,667 m recovery, (**d**) denotes *p* < 0.05 compared to 3,333 m recovery.

There was a significant interaction between time and trial for SPO_2_ (*p* < 0.001). Pre-exercise SPO_2_ values were similar across trials (*p* > 0.05). In all trials SPO_2_ decreased due to exercise (*p* < 0.001). During normoxic exercise participants in the 0 m trial experienced lower SPO_2_ than the 1,667 m (*p* = 0.003), but not the 3,333 m trial (*p* = 0.050) or 5,000 m trial (*p* = 0.186). There was a significant difference between recovery SPO_2_ for all trials (*p* < 0.001), where SPO_2_ decreased with increased hypoxic simulation. Pre-exercise SPO_2_ and recovery SPO_2_ were not different in the 0 m trial (*p* = 0.936). Recovery SPO_2_ within the 1,667 m, 3,333 m, and 5,000 m trials were significantly lower than pre-exercise SPO_2_ (*p* < 0.001). Recovery SPO_2_ was significantly higher (*p* < 0.001) than exercise values in the 0 m trial, not different in the 1,667 m trial (*p* = 0.92), and lower (*p* < 0.001) in the 3,333 m and 5,000 m trials. See Fig. 1 for average SPO_2_ values.

**Fig. 2:**
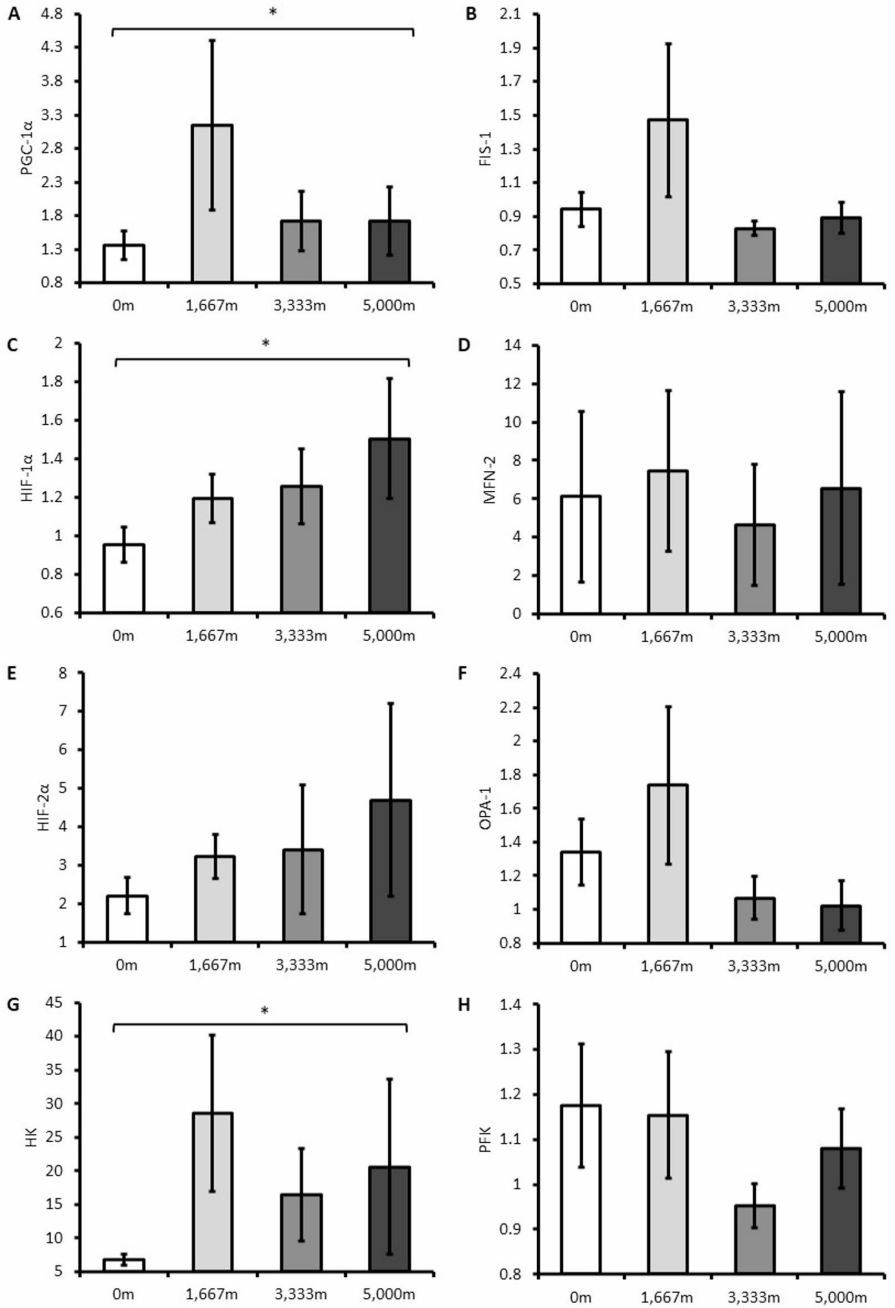
Relative gene expression of (**A**) peroxisome proliferator-activated receptor gamma coactivator 1α (PGC-1α), (**B**) mitochondrial fission 1 protein (FIS-1), (**C**) Hypoxia Inducible Factor 1α (HIF-1α), (**D**) Mitofusin 2 (MFN 2), (**E**) Hypoxia Inducible Factor 2α (HIF-2α), (**F**) Optic Atrophy Gene 1 (OPA-1), (**G**) Hexokinase (HK), (**H**) Phosphofructokinase (PFK) following post- exercise recovery at simulated altitudes of 0 m, 1,667 m, 3,333 m, and 5,000 m. * denotes *p* < 0.05 from pre-exercise.

There was no significant interaction between time and trial for any gene (*p* > 0.05). Gene expression of PGC-1α increased following exercise and 6 h of recovery (*p* = 0.048), but there was no difference between trials (*p* = 0.375) (See Fig. 2A). There was no change in FIS expression following exercise and recovery (*p* = 0.817) and was not different between trials (*p* = 0.274) (See Fig. 2B). HIF-1α gene expression increased following exercise and recovery (*p* = 0.013), but there was no difference between trials (*p* = 0.448) (See Fig. 2C). There was no change in MFN 2 gene expression following exercise and recovery (*p* = 0.121), and was not different between trials (*p* = 0.968) (See Fig. 2D). HIF-2α gene expression did not change following exercise and recovery (*p* = 0.727), and was not different between trials (*p* = 0.081) (See Fig. 2E). There was no change in OPA-1 gene expression following exercise and recovery (*p* = 0.079), and was not different between trials (*p* = 0.325) (See Fig. 2F). HK gene expression increased following exercise and recovery (*p* = 0.014), but there was no difference between trials (*p* = 0.407) (See Fig. 2G). There was no change in PFK gene expression following exercise and recovery (*p* = 0.625) and was not different between trials (*p* = 0.060) (See Fig. 2H).

## Discussion

This work expands on the acute effects of hypoxic recovery on post exercise gene expression. Following 60 min of cycling at 70% Wpeak, recovery in graded simulated altitudes [0 m, 1,667 m, 3,333 m, 5,000 m] was used to investigate the impact of exercise recovery environments on the gene expression of proteins involved in oxygen sensing, metabolism, and mitochondrial dynamics. This model was chosen to replicate patterns used in an acute applied “Live High – Train Low” training strategy similar to the patterns that endurance athletes may use when sleeping in a hypoxic tent. The collected SPO_2_ values from this study confirmed participants were exposed to varying levels of hypoxic stimulus between trials. While we observed changes in gene expression following exercise and recovery, there was no difference in expression between environments. Our study found no indication of a hypoxic threshold up to 5,000 m that would impact gene expression during post exercise hypoxic recovery.

Exercising at sea level and recovering at real or simulated altitude, Live High – Train Low, is commonly used by endurance athletes to attain performance benefits at altitude or at sea level^29,32^. A popular method of Live High – Train Low strategy involves athletes sleeping in hypoxic tents or apartments then training in their typical normoxic environment. However, it is unclear how Live High - Train Low exposures may impact acute adaptive responses to exercise. Alterations in the acute response to exercise may drive chronic training outcomes seen in athletes^2^. Previous research on the acute effect of hypoxic exposure during post exercise recovery on gene expression is limited and has mixed findings^12–14^. When recreationally active males were exposed to a normobaric simulated altitudes of 3,000 m and 4,200 m there was no impact on expression of PGC-1α, HK, PFK, MFN-1, MFN-2, HIF-1α, or cytochrome c oxidase subunit 4^13,14^. However, a reduced gene expression of as low as 0.5 times normoxic response was observed at 5,000 m in PGC-1α, HIF-1α, HIF-2α, MFN-2, and OPA-1^12^. Contrary to these studies, our main findings demonstrated no effect of hypoxia or threshold after which blunting may occur. Possibly indicating indirectly that Live High - Train Low athletes may not experience decrements to mitochondrial biogenesis seen in the chronic exposure of Live High - Train High^37^.

Maximal and submaximal performance at altitude in endurance athletes is impaired compared to sea level^9^, VO_2_max declines at altitude leading to an increased relative intensity for a given activity and increased time to completion in aerobic competition. Leading endurance athletes to acclimate prior to competition to limit performance decrements from hypoxia^29^. Athletes who undergo Live High - Train Low programs not only receive hematological benefits of altitude acclimation but also receive benefits in sea level performance^30,32^. It is important to note that the benefits of altitude training on sea level performance are not definitive^31^. Chronic exposure to hypoxia has been demonstrated to decrease mitochondrial content^20^. However, less is known about the mitochondrial response to other forms of altitude training such as Live High - Train Low protocols. There is research indicating that Live High - Train Low may not reduce mitochondrial content or function in mostly male elite cyclists and triathletes^37^. This was measured using citrate synthase and oxygen flux per mitochondrial unit. While these were not observed in the current study, it may corroborate our findings. In which we observed no acute impact on gene expression, possibly indicating a mechanism in which long term Live High – Train Low protocols do not limit mitochondrial function^2^. This indicates that Live High - Train Low may be an advantageous method for acclimatization, without sacrificing adaptations to exercise at sea level.

The physiological effects of hypoxia can be identified through pulse oximetry, and low SPO_2_ is associated with expression of oxygen sensing genes such as HIF-1α, and HIF-2α^45,46^. In a clinical setting, hypoxia is often defined by a pulse oximeter reading of 92% or lower^47^. While there were significant decreases in SPO_2_ between trials, only recovery during the 3,333 m and 5,000 m trials reached 90% or lower SPO_2_. Typically, HIF-1α is more responsive to acute bouts of hypoxic exposure, while HIF-2α is associated with chronic hypoxic exposure as well as acute hypoxia^48^. In previous research, blunted gene expressions were observed in well trained male participants during recovery at a simulated altitude of 5,000 m with an average pulse oxygen reading of 82.6%^12^. Despite our participants achieving lower SPO_2_ values than observed in previous hypoxia recovery research^12^, 79.6% during recovery in the 5,000 m trial, we did not observe reduced gene expression. In previous studies PGC-1α, HIF-1α, and HIF-2α have been shown to increase in response to 90 min of interval cycling and be blunted by hypoxic recovery at 5,000 m in well trained male participants^12^. Small, but statistically higher expression of oxygen sensing genes PGC-1α and HIF-1α were observed following post-exercise recovery while HIF-2α approached but did not reach significance. Given there was no change in oxygen sensing gene expression with increased simulated altitude it is possible the current hypoxic stimulus was insufficient to detect acute changes. There is some evidence that the muscle can maintain oxygenation even with low SPO_2_^41^ possibly making prolonged or extreme hypoxic stimulus necessary to induce changes in skeletal muscle gene expression. A recent review has also suggested that HIF-1α expression is sensitive to exercise intensity and will not significantly increase following moderate aerobic activity^49^.

Differences in exercise intensity, modality, fitness level, and nutrition have all been demonstrated to alter gene expression response^3–6^. In the current study we observed a relatively minor change in genes such as PGC-1α and PFK compared to a typical exercise response. Differences in exercise methodology across studies could account for variability in the current literature. Expression of certain genes, like PGC-1α and PFK, increases in response to higher exercise intensity intervals compared to steady state training^3,4^. In agreement, minimal change in PGC-1α was observed in a study using the same exercise stimulus as the current study^14^. Previous research in this area utilizing different exercise stimulus, 90 min of interval cycling up to 80% Wpeak or 60 min of continuous cycling at 60% Wpeak, observed changes up to 5- and 15-times baseline for PGC-1α and PFK respectively^12,13^. Given we observed a minimal response in gene expression of PGC-1α, PFK, HIF-1α and HIF-2α to the current exercise protocol, any effect of hypoxic recovery on gene expression may have been obscured. Fitness level could be a confounding variable between studies^50,51^. The average fitness level of our participants, which averaged 4.00 ± 0.51 L·min^− 1^ VO_2_peak, was comparable to other studies which also saw no difference in gene expression^13,14^. However, in a group of male subjects with slightly higher fitness (4.25 ± 0.59 L·min^− 1^) a blunting effect of hypoxic recovery on gene expression was observed on PGC-1α, MFN-2, HIF-1α, and HIF-2α^12^. Another possible confounding factor in previous literature is nutrient intake during recovery, this may have limited gene expression findings between studies by tempering the response to exercise stimulus^6,52^. PFK increased in a similar study that did not include feeding^13^, but not within the current study or another study which included feedings^12^. Indirectly, the lack of response may be due to the inclusion of feeding.

In the current study recreationally active males recovered from exercise for 6 h in all four simulated environments [0 m, 1,667 m, 3,333 m, 5,000 m] and no significant changes in gene expression due to hypoxic exposure during recovery were found in HK, MFN-2, OPA-1, or FIS-1. Gene expression is a multistep process that has different peak expression times depending on the genes involved. In previous literature, when investigating the effect of simulated normobaric hypoxia at 3,000 m or 4,200 m in recreationally and well-trained males, no difference in gene expression due to hypoxia was observed, including MFN-2, PFK or HK^13,14^. However, participants only rested for 4 h following exercise. When a separate group of well-trained males was investigated at 5,000 m, following 6 h of recovery, there was a reduced gene expression response observed in response to hypoxia for genes such MFN-2 and OPA-1^12^. When trained male cyclists lived for 28 days at 3,454 m there was no change observed in expression of MFN-2 or HK^53^. These studies indicate MFN-2 may not respond to hypoxic stimulus in trained individuals. While FIS-1 appears to respond to exercise but not hypoxic stimulus^12,13^. It is possible that differences in timing of the post recovery biopsy could confound findings. While there were no differences between our four simulated environments at 6 h, some genes may have peaked outside of this period. Indeed, following aerobic exercise, running at 75% VO_2_ peak for 65 min, the gene expression of PFK and HK did not peak until 8 to 12 h post exercise^5^. However, It is unclear if hypoxia may alter the time-course of gene expression when compared to normoxic conditions. Lastly, expression of genes such as HIF-1α can influence the expression of other genes such as HK, possibly explaining the observed increase under hypoxic recovery conditions^54^.

The current study serves as proof of concept investigating the acute response of post exercise gene expression to recovery in varying extremes of hypoxic stimulus. As such there are limitations that prevent us from fully elucidating the impact of hypoxic recovery and should be investigated further in future research. The current study only included recreationally active college aged males. This prevents the current findings from being generalized to female athletes. Our participants were also recreationally active and not classically trained endurance athletes, this may have altered observed gene expression response^50,51^. Water was provided ad libitum with no standardization between trials. Therefore, we cannot guarantee that hydration status was constant, which may impact cellular gene expression as demonstrated in liver cells^7^. The response of gene expression in humans even among well documented genes such as PGC-1α is highly variable and limits statistical power of a given study^55^. Additionally, timing of gene expression is complex and varies by gene^5^. For this study we investigated gene expression after 6 h of post exercise recovery because peak activation of gene expression is oftentimes around 4–6 h^5^. While Live High – Train Low regimens incorporate repeated or serial exposures between 12 and 16 h long this study, sought to elucidate a more acute time course of hypoxic recovery on gene expression that might underlie more chronic exposures. Further research should investigate how gene expression is altered over the course of repeated hypoxic exposures. An inherent limitation of gene expression research is the acute nature of gene expression, which may not translate to chronically altered protein content.

## Conclusion

Endurance athletes are expected to perform in a variety of environments from sea level to high altitude. Chronic exposure to altitude can cause detriments to parameters classically associated with sea level endurance performance, such as mitochondrial density and function. Live High -Train Low has been observed to provide the benefits of chronic altitude training without the detriment to the mitochondria^37^. In the current study, which incorporated an acute approximation of the Live High – Train Low protocol, we observed no differences in gene expression related to oxygen sensing and metabolism when acutely recovering from normoxic cycling at graded levels of simulated altitude. Indirectly this could indicate a possible mechanism through which Live High – Train Low protocols for altitude adaptation may provide the benefits of acclimatization while minimizing detrimental impacts.

## Data Availability

Data may be available upon request from the corresponding author, Dr. Dustin Slivka.
